# A rare case of epiploic appendages infarction within an incisional hernia: a usual complain of unusual cause

**DOI:** 10.1093/jscr/rjad483

**Published:** 2023-08-23

**Authors:** Elias Edward Lahham, Qusai A Alsalah, Mohammad I Alsahouri, Abdalrazeq Ghweir, Mohammad AlQadi, Nafez Sarhan

**Affiliations:** Department of Radiation Oncology, Augusta Victoria Hospital, East Jerusalem, Palestinian Authority 9511208, Palestine; Faculty of Medicine, Palestine Polytechnic University, Hebron 150, Palestine; Faculty of Medicine, Palestine Polytechnic University, Hebron 150, Palestine; Faculty of Medicine, Palestine Polytechnic University, Hebron 150, Palestine; General Surgery Department, Beit-Jala Hospital, Bethlehem 4322, Palestine; Palestine Ahliya University, Bethlehem 4322, Palestine

**Keywords:** epiploic appendages infarction, epiploic appendagitis, right lower quadrant painincisional hernia

## Abstract

Epiploic appendagitis (EA) is an uncommon condition caused by infarction of epiploic appendages “small fat outpouchings present on the outside of the colon wall” because of torsion or thrombosis of the main draining vein. It is sometimes misdiagnosed as diverticulitis or appendicitis. Lab tests usually are normal, and the diagnosis is mainly by computerized tomography (CT) scan. Treatment is conservative as it is a self-limited condition, and the symptoms will resolve spontaneously within 2 weeks. However, surgical appendage removal could be necessary if symptoms increase or continue. Here, we report our experience with a 21-year-old male patient, who presented with a 1-day duration of localized right lower quadrant (RLQ) abdominal pain within 18*10 cm incisional hernia, imaging revealed signs of epiploic appendages infarction within the huge incisional hernia. This case describes an atypical scenario for EA, which was successfully managed with surgery. The final pathology report confirms the diagnosis.

## INTRODUCTION

Epiploic appendages are normal peritoneal fat outpouchings on the anti-mesenteric surface of the colon [1], they were described first time by Vesalius in 1543 [2]. An adult colon has between 50 and 100 appendages on average that appear along the colon’s length. Also, the exact role of them is not known [1]. EA represents a rare clinical condition that occurs by an ischemic infarction of an epiploic appendage as a result of torsion or thrombosis of the main draining vein [3, 4]. This condition occurs more frequently than anticipated, but its true prevalence is unknown. EA has been observed in 7% of patients who had initially been suspected of having acute diverticulitis and in 1% of individuals who had initially been suspected of having acute appendicitis [4]. The majority of cases occur during the second to fifth decades, with a mean age of 40 years old with a male-to-female ratio of 4:1 [2, 4]. The chance of developing EA may be increased by obesity and strenuous exercise [4]. Additionally, it is a self-limiting, benign condition [1]. Most individuals are afebrile with normal lab results [5]. CT scan of the abdomen is often used to make the diagnosis [6]. Our study presents a 21-year-old male who presented with localized abdominal pain within a large incisional hernia on the RLQ because of EA that was managed successfully with surgery after the failure of conservative treatment. This report aims to shed light on this rare entity and alerts surgeons not to oversee EA in atypical cases.

## CASE PRESENTATION

**Figure 1:**
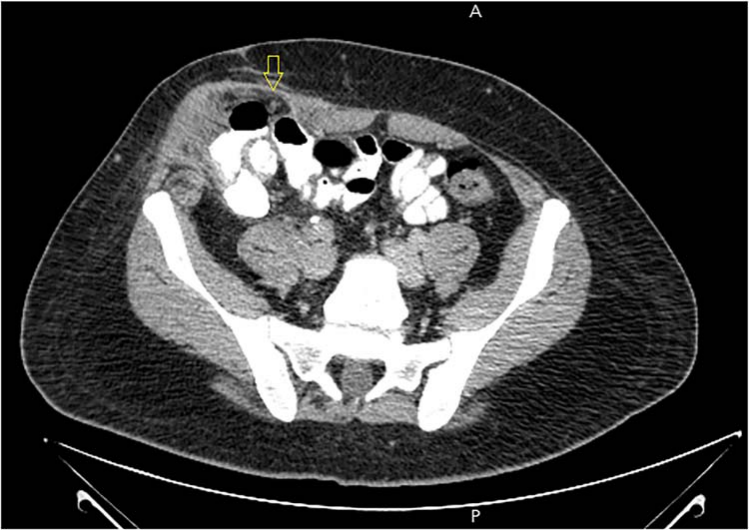
Abdomen CT scan with IV contrast demonstrated a large m peritoneal defect containing cecum and terminal ileum with an oval fatty density 1.5*2 cm represent an inflamed epiploic appendages (arrow) that have a hyper-attenuating rim and “central dot sign” with inflammatory changes in the adjacent fat tissue.

**Figure 2:**
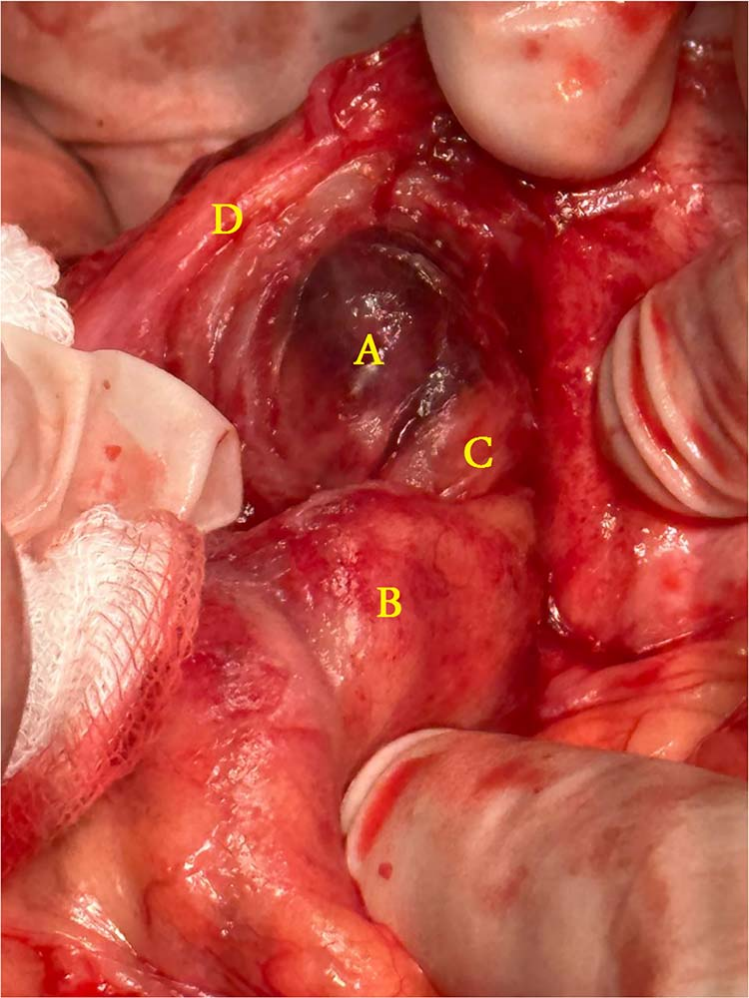
Operative findings: (**A**) infarcted epiploic appendages, (**B**) cecum, (**C**) ascending colon, (**D**) hernial sac.

**Figure 3:**
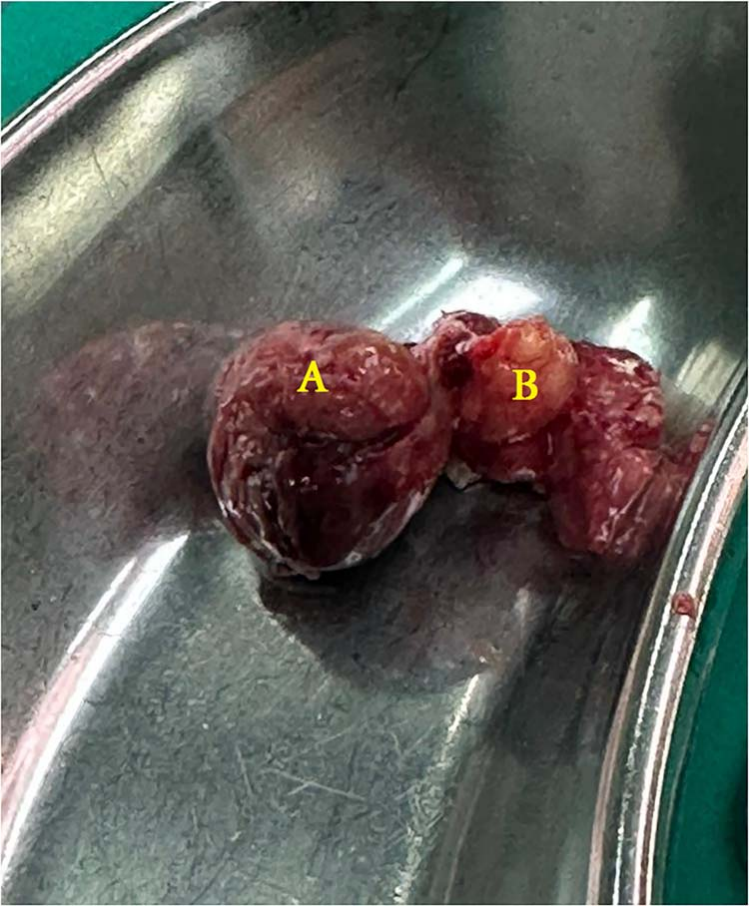
The removed specimen: edematous-infarcted epiploic appendage, measure 3*3 cm (**A**) and a free fatty body (**B**) found in the hernia sac.

A 21-year-old male patient, obese with BMI of 35, known case of incisional hernia that occurred after an open appendectomy one year ago, was admitted to the emergency department complaining of acute abdominal pain for one-day duration. The pain was localized to the RLQ at the site of the incisional hernia, sudden in onset, sharp in character, not radiated with no known aggravating or relieving factors, and not associated with nausea, vomiting, abdominal distention, or change in bowel habit. On admission, he had normal vital signs. Physical examination showed a huge reducible incisional hernia in the RLQ area, at the McBurney scar with no signs of inflammation. There was significant tenderness at the McBurney point. Laboratory tests showed an increase in C-reactive protein (CRP) 180.00 g/l (0–5 g/l) and leukocyte count 17 000*109/L (4.3–10.8 × 109/L). Other Laboratory tests are all within normal. Abdomen CT scan with IV contrast (Fig. 1) demonstrated a large peritoneal defect containing cecum and terminal ileum associated with adjacent facial thickening, prominent lymph nodes, fat stranding, and 1.5*2 cm fatty density, no signs of bowel obstruction. The initial diagnosis consists of EA or early incarcerated hernia. Nasogastric decompression was done, and IV fluids and antibiotics were started. However, the pain gradually increased despite medical management. Because of the severity of the abdominal pain, a decision to proceed with surgical exploration was made. Under general anesthesia, a McBurney incision was done at the site of the incisional hernia. Intraoperatively (Fig. 2), a hernia sac was identified, and multiple aggregated gangrenous EA infarctions were excised (Fig. 3), then a hernioplasty was used to repair the hernial defect. The postoperative period was uneventful. The patient started a soft diet on Day 2 and was discharged from the hospital on Day 4. Histopathologic examination showed an aggregate of fatty-fibrous tissue with fat necrosis, surrounded by active inflammation confirming the diagnosis of EA.

## DISCUSSION

The distribution of epiploic appendages through the colon have a variable frequencies, the most common location is on the rectosigmoid junction, which accounts for 57% of epiploic appendages, whereas the least common location is the descending colon represents 2% [7]. It is 0.5–5 cm long and 1–2 cm thick, it takes blood supply from one or two arteries and drains into a single vein [8]. EA is an epiploic appendage infarction that is caused mainly by torsion or venous thrombosis. Additionally, it may develop secondary to intraperitoneal infections such as appendicitis [9]. EA is a rare disease that occurs predominantly in males [10], with a frequency of 1.3% and an incidence of 8.8 cases per million annually [11]. Obesity, vigorous exercise, and hernia are possible risk factors [12]. Studies showed that obese people have larger epiploic appendages [10]. The clinical presentation is acute, persistent, localized, sharp pain with no radiation [12, 13]. The location of the pain depends on the site of the affected appendage [13]. It occurs mainly in the RLQ region (69%–89%) and the left lower region (8%–16%) [14]. Nausea, vomiting, diarrhea, constipation, and fever are usually absent [12]. Laboratory tests are typically within normal ranges, rarely it associated with mild leukocytosis or elevated CRP [10]. However, our patient was complaining of localized RLQ pain, with elevated white blood cells and CRP. CT scan is the diagnostic test for EA [6], it reveals a 1.5–3.5 cm diameter fat-density ovoid mass surrounded by high attenuation ring known as the hyper-attenuating ring sign [10, 13], with central high attenuation dot corresponding to the thrombosed vein “central dot sign” [6]. Other findings can be seen such as bowel wall thickening and/or peritoneal thickening, which represent the spread of inflammation [6]. On ultrasound, EA presents as a hyper-echoic and noncompressible mass surrounded by a hypo-echoic rim [10, 13]. The treatment is conservative as it is a self-limited condition. And the symptom will resolve spontaneously within 2 weeks [11]. However, the CT finding can persist for 6 months [5]. When conservative treatment fails, surgical excision should be considered [10]. In our cases, the patient was treated by surgical excision of the affected appendage because the pain was severe and not relieved with conservative management.

## CONCLUSION

EA in an incisional hernia is rare and difficult to diagnose. In our case, the patient presented to emergency complaining of acute abdominal pain localized to the incisional hernia without signs of incarcerated or strangulation of the incisional hernia. CT scan results showed the diagnosis of EA, which was effectively treated with surgical resection following unsuccessful conservative treatment. The study’s goal is to provide information on this rare entity and warn surgeons to be aware of the image findings to avoid misdiagnosis.

## Data Availability

The data used to support the findings of this study are included in the article.
